# Higher admission serum total carbon dioxide is independently associated with early seizure recurrence in children with benign convulsions with mild gastroenteritis

**DOI:** 10.3389/fped.2026.1884706

**Published:** 2026-06-16

**Authors:** Ling Zou, Feng Li, Hongyu Li, Zhihong Su

**Affiliations:** Department of Pediatrics, Zigong First People’s Hospital, Zigong, Sichuan, China

**Keywords:** bicarbonates, carbon dioxide, child, preschool, gastroenteritis, risk assessment, seizures

## Abstract

**Background:**

Readily accessible biomarkers for early seizure recurrence in benign convulsions with mild gastroenteritis (CwG) are lacking. We evaluated whether admission serum total carbon dioxide (TCO_2_), a routine venous surrogate for bicarbonate, predicts seizure recurrence within 48 h in children with CwG.

**Methods:**

This retrospective study included children aged 6 months to 6 years diagnosed with CwG (2020–2026). Multivariable logistic regression evaluated the association between admission venous TCO_2_ and seizure recurrence within 48 h, adjusting for age and anticonvulsant use. Model performance underwent bootstrap internal validation. TCO_2_/bicarbonate concordance was assessed in a paired subset.

**Results:**

Of 86 children (median age 14.5 months), 23 (26.7%) experienced recurrence. Median TCO_2_ was higher in the recurrence group (17.4 vs. 15.1 mmol/L, *P* = 0.007). In multivariable analysis, higher TCO_2_ (odds ratio 1.235, 95% confidence interval 1.053–1.448; *P* = 0.009) and younger age (*P* = 0.016) were independently associated with recurrence. The optimism-corrected area under the curve was 0.710, calibration slope was 0.748, and Brier score was 0.164. A TCO_2_ cutoff of 16.25 mmol/L yielded 78.3% sensitivity, 61.9% specificity, and 88.7% negative predictive value. In 29 paired samples, venous TCO_2_ and arterial bicarbonate were strongly correlated (*r* = 0.966, *P* < 0.001).

**Conclusions:**

Higher admission serum TCO_2_ was independently associated with early seizure recurrence in CwG. As a readily accessible venous biomarker, TCO_2_ may represent a candidate marker for bedside risk stratification to guide observation. These findings are hypothesis-generating, and external validation in prospective cohorts is warranted.

## Introduction

Benign convulsions with mild gastroenteritis (CwG), first described by Morooka in 1982 (1), is a well-recognized entity characterized by afebrile seizures in previously healthy young children during acute viral gastroenteritis (2, 3). It predominantly affects children aged 6 months to 3 years and is one of the most common causes of afebrile seizures in this age group, particularly in East Asian populations, although increasing reports have expanded its geographic and ethnic distribution (4, 5). Seizures are typically generalized tonic-clonic, occur without fever, significant dehydration, or marked electrolyte abnormalities, and are associated with favorable long-term neurodevelopmental outcomes; however, seizures may recur in clusters during the acute phase, representing a source of considerable clinical concern (4, 5).

A clinically important feature of CwG is seizure recurrence shortly after presentation. Recurrence rates within the first 24–48 h have been reported at approximately 20%–40% (6, 7), leading to substantial parental anxiety, repeated clinical assessments, potential administration of anticonvulsant agents, and occasionally prolonged emergency department or inpatient observation (6, 7). Several clinical and laboratory risk factors for recurrence have been investigated, including younger age, seizure clustering before presentation, and specific electroencephalographic patterns, but a simple, routinely available biomarker for bedside risk stratification remains desirable (8). Early identification of children at elevated risk at the time of initial presentation would be clinically valuable for guiding monitoring intensity, optimizing resource allocation, and improving anticipatory guidance for families.

The pathogenesis of CwG is incompletely understood (9). Proposed mechanisms include direct viral neurotropism and systemic inflammatory effects, with rotavirus and norovirus being the most frequently implicated pathogens (10, 11). Among the metabolic changes accompanying gastroenteritis, acid–base disturbance may be particularly relevant to seizure susceptibility, as diarrhea-related bicarbonate loss commonly causes metabolic acidosis and concomitant vomiting may introduce competing alkalosis (12). Experimental evidence supports pH-dependent modulation of neuronal excitability and epileptiform activity through multiple converging mechanisms, including modulation of gamma-aminobutyric acid type A receptor function, N-methyl-D-aspartate receptor activity, and acid-sensing ion channel kinetics (13–15). Serum bicarbonate has been highlighted as one of the laboratory parameters most strongly associated with CwG in systematic evidence (16), but direct bicarbonate measurement typically requires blood gas analysis, which is invasive, technically challenging in young infants, and not consistently performed in clinically typical cases.

Serum total carbon dioxide (TCO_2_), a standard component of routine venous biochemistry panels available in virtually all clinical laboratories, predominantly reflects bicarbonate concentration (approximately 95% of the measured value) and serves as a practical and readily accessible surrogate (17–20). However, whether admission TCO_2_ predicts early seizure recurrence in children with CwG remains unknown. We therefore evaluated the association between admission serum TCO_2_ and seizure recurrence within 48 h in children with CwG and assessed whether TCO_2_ was independently associated with recurrence after adjustment for clinically relevant covariates. As a methodological validation step, concordance between TCO_2_ and directly measured bicarbonate was examined in a subset of patients with paired measurements.

## Methods

### Study design and setting

This single-center retrospective observational study was conducted at the First People's Hospital of Zigong, a tertiary care institution in Sichuan Province, China. We reviewed the medical records of all children admitted with a diagnosis of benign convulsions with mild gastroenteritis (CwG) between January 2020 and February 2026. The study was approved by the institutional ethics committee [No. (M) 2026-023], and the requirement for individual informed consent was waived due to the retrospective nature of the study.

### Participants

As CwG lacks a dedicated diagnostic code, candidate records were identified through a keyword search of discharge diagnoses in the hospital electronic medical record system, using “benign convulsion” as the primary search term combined with gastrointestinal diagnostic terms (“enteritis,” “gastrointestinal dysfunction,” and “diarrhea”), for admissions between January 2020 and February 2026. All retrieved records were then manually reviewed by the investigators against the diagnostic criteria of Komori et al., and only those meeting the CwG definition were included. In our institutional system, these diagnoses correspond to ICD-10 codes R56.800 × 005 (benign convulsion), A08/K52.905 (enteritis), K92.901 (gastrointestinal dysfunction), and K52.919 (diarrhea).

Children were included if they met the diagnostic criteria for CwG proposed by Komori et al. (3): (1) age 6 months to 6 years (upper limit set at 6 years for consistency with the diagnostic criteria); (2) the highest recorded body temperature during the illness <38.0 °C, as determined by caregiver-reported maximum temperature and clinical measurement upon hospital arrival; (3) occurrence of afebrile generalized seizures during the course of acute mild gastroenteritis; (4) no severe dehydration or seizure-inducing electrolyte abnormalities; (5) no prior history of epilepsy or febrile seizures and no neurodevelopmental abnormalities; and (6) normal neurological examination between seizures. Dehydration was assessed using WHO criteria, and patients with moderate-to-severe dehydration were excluded.

Lumbar puncture, electroencephalography (EEG), and cranial computed tomography (CT) were performed at the discretion of the attending physician in cases with recurrent, prolonged, or atypical seizures, signs of meningeal irritation, or diagnostic uncertainty. In patients who did not undergo cerebrospinal fluid examination, the absence of central nervous system infection was inferred from the benign clinical course, including spontaneous resolution of seizures, normal interictal neurological findings, and uneventful clinical recovery. Patients with identified structural brain lesions, metabolic disorders unrelated to gastroenteritis, or incomplete records were excluded.

### Data collection

We extracted demographic variables (age, sex, weight), gastroenteritis features (vomiting and diarrhea episodes in the 24 h before admission; dehydration grade), and seizure-related variables (number of pre-arrival seizures; interval from the last pre-admission seizure to blood sampling). Pre-sampling treatments (anticonvulsant medication and intravenous fluids) and length of hospital stay were recorded. Pre-arrival seizure count was additionally dichotomized as ≥2 vs. <2, as the occurrence of multiple seizures before presentation may indicate a higher propensity for seizure clustering (6).

### Laboratory measurements

Venous blood samples were collected at admission as part of routine care. Whenever feasible, samples were obtained within approximately 30 min of arrival and before therapeutic intervention; if anticonvulsant medications and/or intravenous fluids were administered before sampling due to clinical necessity, this was recorded and accounted for in analyses. Serum total carbon dioxide (TCO_2_) was measured as a standard component of the routine admission venous biochemistry panel (which includes liver function, renal function, electrolytes, and cardiac enzymes) using a phosphoenolpyruvate carboxylase (PEPC) enzymatic method on an automated biochemistry analyzer (LABOSPECT 008, Hitachi High-Tech Corporation, Tokyo, Japan), without the need for additional testing. Sodium, chloride, potassium, calcium, glucose, blood urea nitrogen, creatinine, and uric acid were measured from the same specimen. In a subset of patients, arterial blood gas analysis was performed using the GEM Premier 3,500 analyzer (Instrumentation Laboratory, Bedford, MA, USA), and bicarbonate ($\mathrm{HC}O_{3}^{-}$) was recorded for concordance assessment. Stool rotavirus and adenovirus antigens were detected using a latex agglutination assay; norovirus testing was not routinely available at our institution during the study period. The TCO_2_ assay and CwG diagnostic criteria were unchanged throughout the study period.

### Outcome definition

The primary outcome was seizure recurrence within 48 h of admission. The 48-hour window was prespecified based on prior reports indicating that seizure recurrences in CwG typically cluster within the first 24–48 h (7). Seizure recurrence was defined as any clinically observed generalized convulsive episode after admission documented by the attending medical and nursing staff. Patients were monitored by routine ward-based observation, and the entire hospitalization record was reviewed to capture all recurrence events.

### Statistical analysis

Continuous variables are presented as median (interquartile range) and compared using the Mann–Whitney *U*-test. Categorical variables are presented as *n* (%) and compared using the chi-square test or Fisher's exact test, as appropriate. In patients with paired measurements, concordance between TCO_2_ and $\mathrm{HC}O_{3}^{-}$ was assessed using Pearson and Spearman correlations and Bland–Altman analysis (mean difference and 95% limits of agreement) (21). Bland–Altman agreement analysis included all paired observations, with no prespecified rule for outlier exclusion; exclusion of any single observation is reported only as a *post-hoc* sensitivity analysis, and the primary agreement estimates are based on the full set of paired samples.

The association between admission TCO_2_ and seizure recurrence was evaluated using multivariable logistic regression. Model specifications were: M1, TCO_2_ only; M2, TCO_2_ plus pre-arrival seizure count, seizure-to-sampling interval, and anticonvulsant medication indicators (midazolam and phenobarbital; reference: none); M3 (final), TCO_2_ plus age (months) and anticonvulsant medication indicators. M2 adjusted for seizure-related variables and M3 for demographic and treatment variables. Given 23 recurrence events, the number of predictors in the primary model was restricted to four to maintain an events-per-variable ratio of approximately 5. An extended sensitivity model (M_extended) additionally included pre-arrival seizure count and seizure-to-sampling interval. An exploratory model (M_exploratory) further included serum chloride and pre-sampling intravenous fluids.

Model performance was assessed using the omnibus likelihood ratio test, Hosmer–Lemeshow test, Nagelkerke R^2^, and area under the receiver operating characteristic curve (AUC) with 95% confidence intervals (22, 23). Optimal cutoffs for TCO_2_ and M3-predicted probability were determined using the Youden index. Calibration was assessed by grouping predicted probabilities into deciles and visualized with a calibration plot. Internal validation of M3 was performed using bootstrap resampling (1,000 resamples) in R version 4.5.3 with the rms package to obtain optimism-corrected AUC, calibration slope, calibration intercept, and Brier score. Multicollinearity in M3 was assessed using variance inflation factors (VIF), with VIF > 5 indicating potential collinearity. A prespecified sensitivity analysis repeated M3 after excluding patients who received intravenous fluids before sampling. Receiver operating characteristic curves and related figures were plotted using GraphPad Prism version 10.6.0 for Windows (GraphPad Software, San Diego, CA, USA); the model calibration curve was generated with the rms package in R (version 4.5.3) as part of bootstrap internal validation.

All analyses were performed using IBM SPSS Statistics 27.0 (IBM Corporation, Armonk, NY, USA) unless otherwise specified. No imputation was performed. Two-tailed *P* < 0.05 was considered statistically significant.

## Results

### Study population

A total of 86 children met the diagnostic criteria for CwG and were included in the analysis. Of these, 23 (26.7%) experienced seizure recurrence after hospital admission and 63 (73.3%) did not. All 23 recurrence events occurred within the first 24 h of admission, with no further seizure episodes documented between 24 h and discharge in any patient.

### Baseline characteristics

Baseline demographic, clinical, and laboratory characteristics are shown in Table 1. The overall median age was 14.5 months [interquartile range (IQR), 12.0–24.0], with ages ranging from 7 to 67 months, and a slight male predominance (53.5%). The median serum TCO_2_ was 16.2 mmol/L (IQR, 13.3–17.9).

**Table 1:** Baseline demographic, clinical, and laboratory characteristics of children with benign convulsions with mild gastroenteritis, stratified by seizure recurrence status.

Variable	Total (*n* = 86)	No recurrence (*n* = 63)	Recurrence (*n* = 23)	*P* value
Demographics				
Age, months	14.5 (12.0–24.0)	16.0 (12.0–24.0)	12.0 (12.0–19.0)	0.031
Male sex, *n* (%)	46 (53.5)	33 (52.4)	13 (56.5)	0.733
Body weight, kg	10.5 (9.5–12.0)	10.5 (9.5–12.0)	10.0 (9.5–11.9)	0.736
Gastroenteritis characteristics				
Vomiting episodes per 24 h	3 (1–4)	3 (0–4)	3 (1–5)	0.516
Diarrhea episodes per 24 h	4 (3–5)	4 (3–5)	4 (2–5)	0.203
Dehydration grade, *n* (%)				0.757
None	50 (58.1)	36 (57.1)	14 (60.9)	
Mild	36 (41.9)	27 (42.9)	9 (39.1)	
Stool viral test, *n* (%)				0.187*
Negative	77 (89.5)	58 (92.1)	19 (82.6)	
Rotavirus positive	8 (9.3)	5 (7.9)	3 (13.0)	
positive	1 (1.2)	0 (0.0)	1 (4.3)	
Seizure characteristics				
Pre-arrival seizure count	1 (1–2)	1 (1–1)	1 (1–2)	0.238
Pre-arrival seizures ≥ 2, *n* (%)	25 (29.1)	15 (23.8)	10 (43.5)	0.075
Last seizure-to-sampling, min	97 (70–176)	98 (80–173)	85 (56–186)	0.219
Anticonvulsant administered, *n* (%)				0.220
None	31 (36.0)	22 (34.9)	9 (39.1)	
Midazolam	14 (16.3)	8 (12.7)	6 (26.1)	
Phenobarbital	41 (47.7)	33 (52.4)	8 (34.8)	
IV fluids before sampling, *n* (%)	16 (18.6)	14 (22.2)	2 (8.7)	0.216
Laboratory values				
Serum TCO_2_, mmol/L	16.2 (13.3–17.9)	15.1 (12.9–17.5)	17.4 (16.3–19.3)	0.007
Sodium, mmol/L	134.5 (133.2–135.8)	134.1 (133.1–135.6)	135.1 (134.0–136.1)	0.103
Chloride, mmol/L	100.2 (97.7–102.6)	100.4 (98.0–102.3)	99.9 (97.4–103.0)	0.781
Potassium, mmol/L	4.36 (4.07–4.66)	4.38 (4.09–4.65)	4.30 (3.86–4.70)	0.432
Calcium, mmol/L	2.37 (2.30–2.43)	2.36 (2.30–2.44)	2.38 (2.30–2.42)	0.769
Glucose, mmol/L	4.02 (3.62–4.92)	4.10 (3.73–5.11)	3.92 (3.35–4.20)	0.100
BUN, mmol/L	4.20 (3.17–5.15)	4.19 (2.98–5.20)	4.20 (3.40–4.94)	0.977
Creatinine, μmol/L	22.2 (18.8–26.2)	21.8 (18.5–25.5)	24.0 (18.8–28.2)	0.230
Uric acid, μmol/L	514 (389–636)	508 (390–634)	528 (385–653)	0.841
Outcome				
Length of stay, days	4 (3–5)	4 (3–5)	4 (3–6)	0.507

Continuous variables are presented as median (interquartile range) and compared using the Mann–Whitney *U*-test. Categorical variables are presented as *n* (%) and compared using the chi-square test or Fisher's exact test, as appropriate. Bold *P* values indicate statistical significance at *P* < 0.05. Multiple comparisons in Table 1 were performed for descriptive purposes without adjustment for multiplicity.

* *P*-value calculated using Fisher's exact test due to small expected cell counts.

BUN, blood urea nitrogen; IV, intravenous; TCO_2_, total carbon dioxide.

In univariable comparisons, serum TCO_2_ was significantly higher in the recurrence group than in the non-recurrence group [median 17.4 (IQR, 16.3–19.3) vs. 15.1 (IQR, 12.9–17.5) mmol/L; *P* = 0.007], and age was significantly lower in the recurrence group [median 12.0 (IQR, 12.0–19.0) vs. 16.0 (IQR, 12.0–24.0) months; *P* = 0.031]. A numerically higher proportion of children in the recurrence group had ≥2 pre-arrival seizures (43.5% vs. 23.8%; *P* = 0.075), although this difference did not reach statistical significance at the prespecified threshold. Age was not significantly correlated with serum TCO_2_ in the overall cohort (Spearman *ρ* = 0.084, *P* = 0.440).

No significant between-group differences were observed in sex, body weight, vomiting/diarrhea frequency, dehydration grade, anticonvulsant medication, pre-sampling intravenous fluids, or other measured laboratory parameters (all *P* > 0.05; Table 1). Stool viral testing identified rotavirus in 8 patients (9.3%) and adenovirus in 1 patient (1.2%), while 77 patients (89.5%) tested negative for both pathogens, with no significant difference in distribution between groups (*P* = 0.187, Fisher's exact test).

Lumbar puncture was performed in 5 patients (5.8%), and cerebrospinal fluid results were unremarkable in all cases examined. EEG was performed in 41 patients (47.7%) and cranial CT in 22 (25.6%). EEG and CT were more frequently obtained in the recurrence group (EEG: 78.3% vs. 36.5%, *P* = 0.001; CT: 56.5% vs. 14.3%, *P* < 0.001), reflecting additional diagnostic evaluation prompted by seizure recurrence. Among the 41 patients who underwent EEG, 5 showed diffuse background slowing and 1 had findings suggestive of epileptiform activity; the remaining 35 were normal. Cranial CT findings were unremarkable in all 22 cases examined.

### Concordance between serum TCO_2_ and directly measured bicarbonate

**Figure 1:**
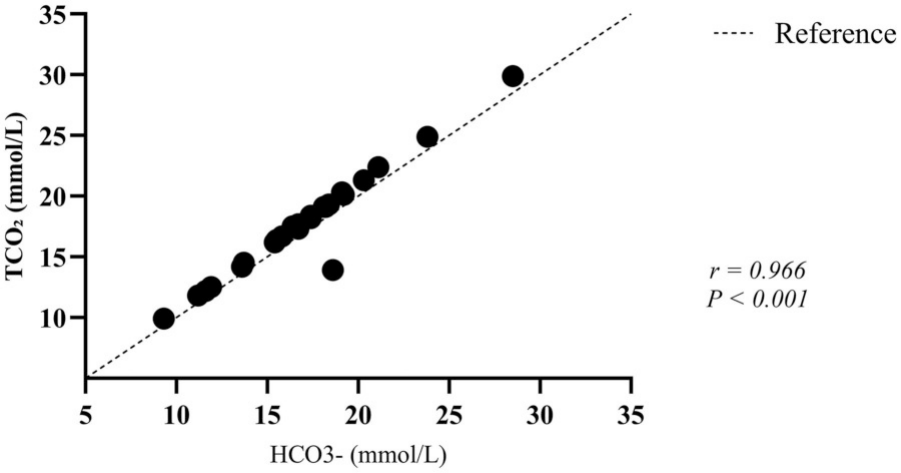
Scatter plot demonstrating the concordance between serum total carbon dioxide (TCO_2_) and directly measured bicarbonate ($\mathrm{HC}O_{3}^{-}$). The plot displays paired measurements from 29 children with benign convulsions with mild gastroenteritis. Serum TCO_2_ was measured by an automated venous biochemistry analyzer, and serum $\mathrm{HC}O_{3}^{-}$ was measured concurrently by an arterial blood gas analyzer. The solid black line represents the linear regression fit (Pearson *r* = 0.966, *P* < 0.001), and the dashed gray line represents the line of identity (y = x), indicating perfect agreement. $\mathrm{HC}O_{3}^{-}$, bicarbonate; TCO_2_, total carbon dioxide.

Twenty-nine patients (33.7%) underwent concurrent arterial blood gas analysis, providing paired measurements. Compared with those without blood gas testing, these patients had higher TCO_2_ levels (*P* = 0.001) and a higher recurrence rate (48.3% vs. 15.8%, *P* = 0.001), while age did not differ (*P* = 0.803), suggesting that blood gas analysis was preferentially performed in patients with more clinically concerning presentations. In this subset, the mean TCO_2_ was 17.83 ± 4.08 mmol/L and the mean HCO₃⁻ was 17.12 ± 3.84 mmol/L. TCO_2_ and HCO₃⁻ were strongly correlated (Pearson r = 0.966; Spearman *ρ* = 0.916; both *P* < 0.001; Figure 1). Bland–Altman analysis showed a mean difference (TCO_2_−HCO₃⁻) of 0.71 ± 1.06 mmol/L with 95% limits of agreement from −1.37 to 2.79 mmol/L; 28 of 29 observations (96.6%) lay within the limits of agreement (Supplementary Figure S1). One observation showed a markedly discordant difference of −4.70 mmol/L. In a *post-hoc* sensitivity analysis excluding this single outlying observation (*n* = 28), the Pearson correlation coefficient exceeded 0.999, the mean difference was 0.90 ± 0.22 mmol/L, and the 95% limits of agreement narrowed to 0.47–1.33 mmol/L. Given the retrospective design, the specific reason for this outlying observation (e.g., a time difference between the two samplings or specimen-handling factors) could no longer be ascertained; the primary agreement conclusions are therefore based on all 29 paired samples, and this exclusion is presented only as a supplementary observation.

### Association between serum TCO_2_ and seizure recurrence

Logistic regression results are summarized in Table 2. In M1, higher TCO_2_ was associated with increased odds of recurrence [odds ratio (OR) 1.169 per 1 mmol/L, 95% confidence interval (CI) 1.017–1.345; *P* = 0.028]. The association remained significant in M2 (OR 1.158, 95% CI 1.005–1.335; *P* = 0.043), while none of the additional covariates reached significance. In the final model (M3), TCO_2_ (OR 1.235, 95% CI 1.053–1.448; *P* = 0.009) and younger age (OR 0.900 per month, 95% CI 0.826–0.980; *P* = 0.016) were independently associated with recurrence. Neither midazolam (OR 1.782, 95% CI 0.429–7.407; *P* = 0.426) nor phenobarbital (OR 0.571, 95% CI 0.172–1.900; *P* = 0.361) reached statistical significance.

**Table 2:** Multivariable logistic regression models for seizure recurrence within 48 h in children with benign convulsions with mild gastroenteritis (*n* = 86).

Variable	M1 OR (95% CI)	P	M2 OR (95% CI)	P	M3 OR (95% CI)	P
TCO_2_, per 1 mmol/L	1.169 (1.017–1.345)	0.028	1.158 (1.005–1.335)	0.043	1.235 (1.053–1.448)	0.009
Age, per month	—	—	—	—	0.900 (0.826–0.980)	0.016
Pre-arrival seizure count	—	—	1.240 (0.531–2.895)	0.619	—	—
Seizure-to-sampling interval, per min	—	—	0.998 (0.991–1.004)	0.498	—	—
Midazolam vs. none	—	—	1.447 (0.312–6.698)	0.637	1.782 (0.429–7.407)	0.426
Phenobarbital vs. none	—	—	0.652 (0.204–2.076)	0.469	0.571 (0.172–1.900)	0.361
Model performance						
Omnibus LR *χ*^2^ (P)	5.407 (0.020)		8.007 (0.156)		15.696 (0.003)	
Nagelkerke R^2^	0.089		0.129		0.243	
Hosmer–Lemeshow P	0.614		0.447		0.572	

M1: unadjusted (TCO_2_ only). M2: TCO_2_ adjusted for pre-arrival seizure count, seizure-to-sampling interval, and anticonvulsant medication. M3 (final): TCO_2_ adjusted for age and anticonvulsant medication. Bold *P*-values indicate *P* < 0.05. The reference category for anticonvulsant medication is no medication.

CI, confidence interval; LR, likelihood ratio; OR, odds ratio; TCO_2_, total carbon dioxide.

### Model performance and sensitivity analyses

**Figure 2:**
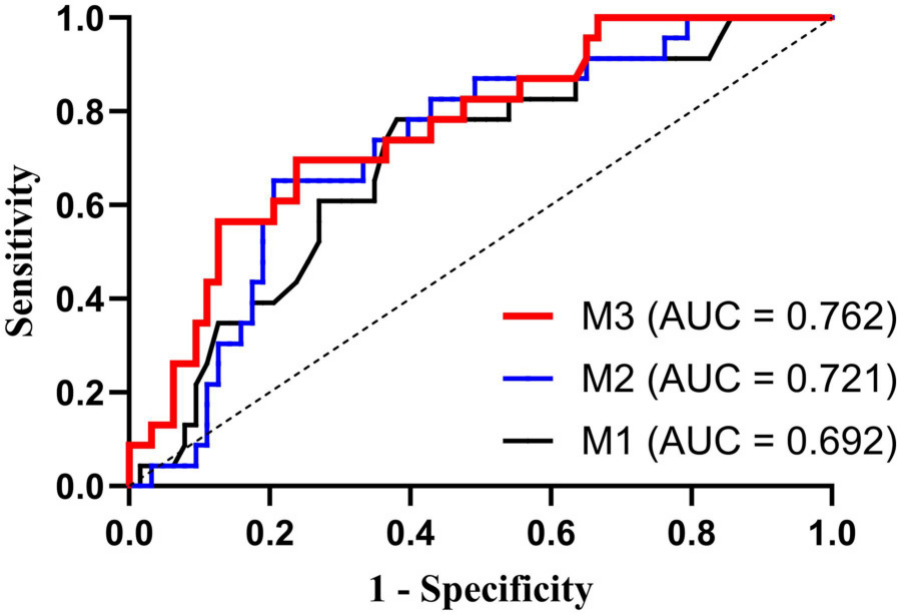
Receiver operating characteristic (ROC) curves for the prediction of seizure recurrence within 48 h. Three multivariable logistic regression models are displayed: M1 (serum TCO_2_ alone; AUC = 0.692, 95% CI 0.570 to 0.813), M2 (TCO_2_ adjusted for pre-arrival seizure count, seizure-to-sampling interval, and anticonvulsant medication; AUC = 0.721, 95% CI 0.606 to 0.836), and M3 (the final model: TCO_2_ adjusted for age and anticonvulsant medication; AUC = 0.762, 95% CI 0.652 to 0.871). The diagonal dashed line represents the reference line of no discrimination (AUC = 0.50). AUC, area under the curve; CI, confidence interval; TCO_2_, total carbon dioxide.

Discrimination improved from M1 to M3 (AUC 0.692, 0.721, and 0.762, respectively; Figure 2). The Hosmer–Lemeshow goodness-of-fit test for M3 was non-significant (*P* = 0.572), and the Nagelkerke R² was 0.243. Bootstrap internal validation (1,000 resamples) yielded an optimism-corrected AUC of 0.710 for the final model; optimism-corrected calibration slope 0.748; calibration intercept −0.194; and Brier score 0.164 (Supplementary Figure S2). Collinearity diagnostics for M3 revealed variance inflation factors of 1.057 (TCO_2_), 1.060 (age), 1.226 (midazolam), and 1.263 (phenobarbital), all well below the threshold of 5.

Using the Youden index, the optimal cutoff for serum TCO_2_ was 16.25 mmol/L (sensitivity 78.3%, specificity 61.9%, positive predictive value 42.8%, negative predictive value 88.7%). For the M3 predicted probability, the optimal cutoff was 0.317 (sensitivity 69.6%, specificity 76.2%, positive predictive value 51.6%, negative predictive value 87.3%).

In sensitivity analyses, TCO_2_ remained independently significant in the extended model (M_extended; OR 1.254, 95% CI 1.062–1.482; *P* = 0.008) and after excluding patients who received intravenous fluids before sampling (OR 1.328, 95% CI 1.100–1.603; *P* = 0.003; Table 3). In the blood gas subset (*n* = 29), lactate was not significantly correlated with serum TCO_2_ (*r* = −0.181, *P* = 0.347) or bicarbonate (*r* = −0.178, *P* = 0.365), and no statistically significant between-group differences were detected in pH, PCO_2_, base excess, or lactate, although the small sample size limited statistical power for these comparisons. In this subset, TCO_2_ was correlated with PCO_2_ (*r* = 0.821, *P* < 0.001) and base excess (*r* = 0.883, *P* < 0.001). In an exploratory analysis (M_exploratory), intravenous fluid administration before sampling was associated with lower odds of recurrence; given the small number of treated recurrence events (*n* = 2), this finding is reported as hypothesis-generating (Supplementary Table S1).

**Table 3:** Sensitivity analysis: logistic regression model (M3 specification) after exclusion of patients who received intravenous fluids prior to blood sampling (*n* = 70).

**Variable**	**OR (95% CI)**	***P* value**
TCO_2_, per 1 mmol/L	1.328 (1.100–1.603)	0.003
Age, per month	0.892 (0.814–0.977)	0.014
Midazolam vs. none	3.046 (0.483–19.219)	0.236
Phenobarbital vs. none	0.643 (0.182–2.278)	0.494
Model performance		
Omnibus LR chi-square (P)	17.021 (0.002)	
Nagelkerke R-squared	0.306	
Hosmer-Lemeshow P	0.434	

Bold values indicate statistical significance at *P* < 0.05. The analysis included 70 patients (49 without recurrence, 21 with recurrence) after exclusion of 16 patients who had received intravenous fluids before blood sampling. With 21 recurrence events and 4 predictors, the events-per-variable ratio in this subgroup was 5.25; estimates should be interpreted as supportive.

CI, confidence interval; LR, likelihood ratio; OR, odds ratio; TCO_2_, total carbon dioxide.

## Discussion

In this single-center retrospective cohort of 86 children with CwG, higher admission serum TCO_2_ measured on routine venous biochemistry was independently associated with seizure recurrence after adjustment for age and anticonvulsant medication type. To our knowledge, this is the first study to identify TCO_2_, a readily accessible venous biomarker, as a candidate predictor of early seizure recurrence in CwG. Although the outcome window was prespecified as 48 h based on prior literature 6), all recurrences occurred within the first 24 h, consistent with the clustered nature of CwG seizures (6, 7) and suggesting that the highest-risk period is concentrated early after presentation. Future studies should evaluate whether a 24-hour monitoring window provides equivalent clinical information.

The odds ratio for TCO_2_ increased slightly after adjustment for age in M3. Age was not significantly correlated with TCO_2_ in our cohort, indicating limited confounding between these variables; the modest change in effect size across models may reflect the non-collapsibility of odds ratios in logistic regression rather than a true suppression effect. This underscores the importance of interpreting effect size changes across models cautiously. In logistic regression, conditional (adjusted) odds ratios can differ from marginal (unadjusted) odds ratios even without confounding, because the odds ratio is a non-linear measure.

Regardless of mechanism, the directionality of the association merits consideration: higher TCO_2_, reflecting a less acidotic systemic milieu, was linked to greater recurrence risk. Although causal inference is not possible in this observational study, the finding is compatible with pH-dependent modulation of neuronal excitability. Experimental work has demonstrated that lowering extracellular pH suppresses epileptiform activity (24), that protons inhibit N-methyl-D-aspartate receptors (25), and that neuronal activity dynamically modulates local pH (26); clinically, carbonic anhydrase inhibitors exploit acidosis as an antiseizure mechanism (27). Together with evidence on GABA receptor signaling and acid-sensing ion channels (13–15), these observations provide a plausible framework linking systemic acid–base status to seizure susceptibility, although direct application to CwG remains speculative.

A practical advantage of TCO_2_ is its availability from routine venous biochemistry, whereas blood gas testing is invasive and not consistently performed in clinically typical cases. Prior evidence, including a meta-analysis by Miyagi et al., highlighted bicarbonate-related abnormalities as characteristic laboratory findings in CwG (16). In the paired subset, TCO_2_ and arterial blood gas bicarbonate showed strong concordance, supporting the use of venous TCO_2_ as a pragmatic surrogate. Notably, blood gas testing was preferentially performed in patients with higher TCO_2_ levels and higher recurrence rates, indicating selection in this subset and limiting generalizability of agreement estimates to the broader CwG population. Formal concordance studies between venous TCO_2_ and blood gas bicarbonate in pediatric gastroenteritis populations have not been previously reported; our findings provide initial data supporting the interchangeability of these measurements in this clinical context.

The absolute between-group difference in TCO_2_ was modest (approximately 2.3 mmol/L) but occurred in a cohort with overall low TCO_2_ levels (cohort median 16.2 mmol/L), consistent with bicarbonate loss during gastroenteritis. In this metabolic context, a shift of this magnitude may represent a physiologically meaningful change in acid–base status relative to the seizure threshold. Nevertheless, because both group medians fell within the acidotic range and the interquartile ranges overlapped substantially, this difference is too small to serve as a stand-alone discriminator at the individual-patient level; at the 16.25 mmol/L cutoff the specificity (61.9%) and positive predictive value (42.8%) were limited, whereas the high negative predictive value (88.7%) indicates that the principal clinical value of TCO_2_ lies in helping to identify lower-risk children (a rule-out application) rather than in confirming high risk. TCO_2_ should therefore be interpreted as one component of an overall clinical assessment rather than as an isolated decision threshold. Postictal lactic acidosis is an alternative contributor to acid–base changes (28); however, the seizure-to-sampling interval did not differ between groups, and in the blood gas subset lactate was not correlated with TCO_2_ or bicarbonate. While these subset analyses were underpowered, they do not support lactate-driven postictal acidosis as the primary explanation for the observed association.

Younger age was independently associated with recurrence, consistent with prior reports (29, 30) and with age-dependent susceptibility to acute symptomatic seizures, which may reflect developmental maturation of inhibitory neural circuits (31, 32). In contrast, uric acid–often reported as elevated in CwG (12, 16)–did not differ between recurrence groups in our cohort, suggesting that TCO_2_ and uric acid may reflect different aspects of the acute metabolic response. The pre-arrival seizure count showed a non-significant trend toward higher values in the recurrence group (*P* = 0.075), consistent with its established role as a recurrence predictor in larger studies (30); the lack of significance in our study likely reflects limited statistical power.

Neither midazolam nor phenobarbital was independently associated with recurrence in the final model. This likely reflects limited power and confounding by indication, as medication choice and timing are influenced by seizure severity and clinical context. Larger prospective studies are needed to clarify whether acute antiseizure medication selection modifies short-term recurrence risk in CwG.

From a clinical perspective, model discrimination was moderate (AUC 0.762; optimism-corrected AUC 0.710) with moderate overfitting (optimism-corrected calibration slope 0.748). The optimism-corrected calibration slope below 1.0 indicates that predicted probabilities at the extremes are somewhat too spread out, which is expected given the limited events-per-variable ratio and underscores the need for external validation (33, 34). The high negative predictive value at the TCO_2_ cutoff (approximately 88%) suggests that the model may be most useful for identifying children at low risk of recurrence who could be candidates for shorter observation periods and earlier discharge. For example, a 14-month-old child presenting with CwG and a TCO_2_ of 18 mmol/L who has not received anticonvulsant medication would have a model-predicted recurrence probability of approximately 44%, exceeding the optimal probability threshold of 0.317 and suggesting the need for extended monitoring and proactive parental counseling. This example is intended to illustrate model application and should not be used for clinical decision-making until the model has been externally validated. Given the single-center retrospective design, the limited number of recurrence events (events-per-variable ratio ≈ 5.75), and the absence of external validation, these findings should be regarded as hypothesis-generating, and the value of TCO_2_ as a predictor warrants confirmation in larger external cohorts.

Finally, the rotavirus detection rate in our cohort (9.3%) was lower than rates reported in earlier CwG studies (30%–70%) (5, 10), likely reflecting the post-rotavirus-vaccination era and the temporal shift toward norovirus as the predominant pathogen (5). Norovirus testing was unavailable at our institution, limiting pathogen-specific analyses. Given that different pathogens may vary in neurotropic potential and metabolic effects, systematic viral identification in future studies would improve etiologic resolution and external validity (5, 11).

### Limitations

This retrospective single-center study has limitations that may affect generalizability. The number of recurrence events was modest (*n* = 23), yielding an events-per-variable ratio of 5.75 in the primary model; although bootstrap internal validation was performed, external validation in larger independent cohorts is needed to confirm model stability. Accordingly, the present findings should be interpreted as hypothesis-generating rather than as establishing TCO_2_ as a validated predictor. Seizure recurrence was determined by clinical observation without continuous EEG monitoring; missed subclinical events would be expected to bias associations toward the null. In addition, a proportion of patients received anticonvulsant medication and/or intravenous fluids before blood sampling because of clinical necessity, which may have altered the measured TCO_2_ value; although we addressed this through a prespecified sensitivity analysis excluding patients given intravenous fluids before sampling, residual influence on the biomarker cannot be excluded. Because anticonvulsant agents and fluids were preferentially administered to children with more severe presentations, the apparent lack of association between these treatments and recurrence is susceptible to confounding by indication and should not be interpreted as evidence of ineffectiveness. Furthermore, the identification of the initial seizure, the afebrile status, and the pre-arrival seizure count relied in part on caregiver observation and report, which may be subject to recall and ascertainment bias. Lumbar puncture was performed in only 5.8% of patients; in the remainder, the absence of central nervous system infection was inferred from the benign clinical course, which may not entirely exclude subclinical meningitis. Viral testing was limited to rotavirus and adenovirus, and norovirus was unavailable, precluding pathogen-specific analyses. Serial acid–base measurements were not routinely obtained, precluding assessment of dynamic metabolic trajectories. The study period overlapped with the COVID-19 pandemic, which may have influenced gastroenteritis epidemiology and healthcare-seeking behavior.

## Conclusions

Higher admission serum TCO_2_ was independently associated with early seizure recurrence in children with CwG, and this association was consistent across multiple models and sensitivity analyses. Venous TCO_2_ showed strong concordance with arterial blood gas bicarbonate and may represent a candidate biomarker for bedside risk stratification. However, given the limited sample size and the absence of external validation, these findings should be regarded as hypothesis-generating. Prospective multicenter studies with comprehensive viral pathogen identification, external validation, and serial acid–base monitoring are warranted to confirm the prognostic value of TCO_2_ and to determine whether TCO_2_-based risk stratification can improve clinical management of CwG.

## Data Availability

The raw data supporting the conclusions of this article will be made available by the authors, without undue reservation.
