# Supplementary figures and images for: Higher admission serum total carbon dioxide is independently associated with early seizure recurrence in children with benign convulsions with mild gastroenteritis
Source: Front Pediatr. 2026 Jun 16;14:1884706. doi: 10.3389/fped.2026.1884706 (PMC13314874; doi:10.3389/fped.2026.1884706)

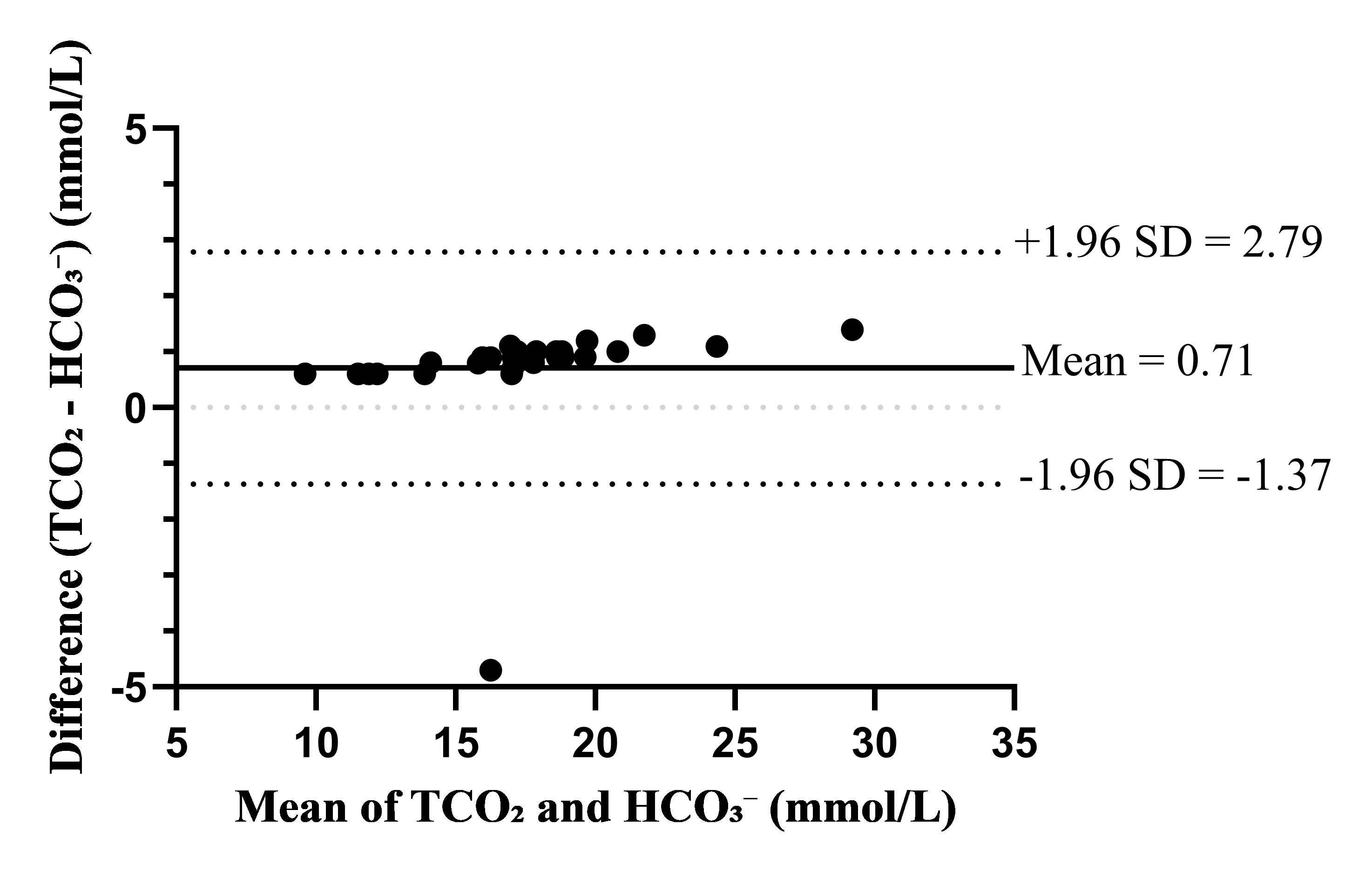

Supplement: Supplementary file 1 [file Image1.tif]

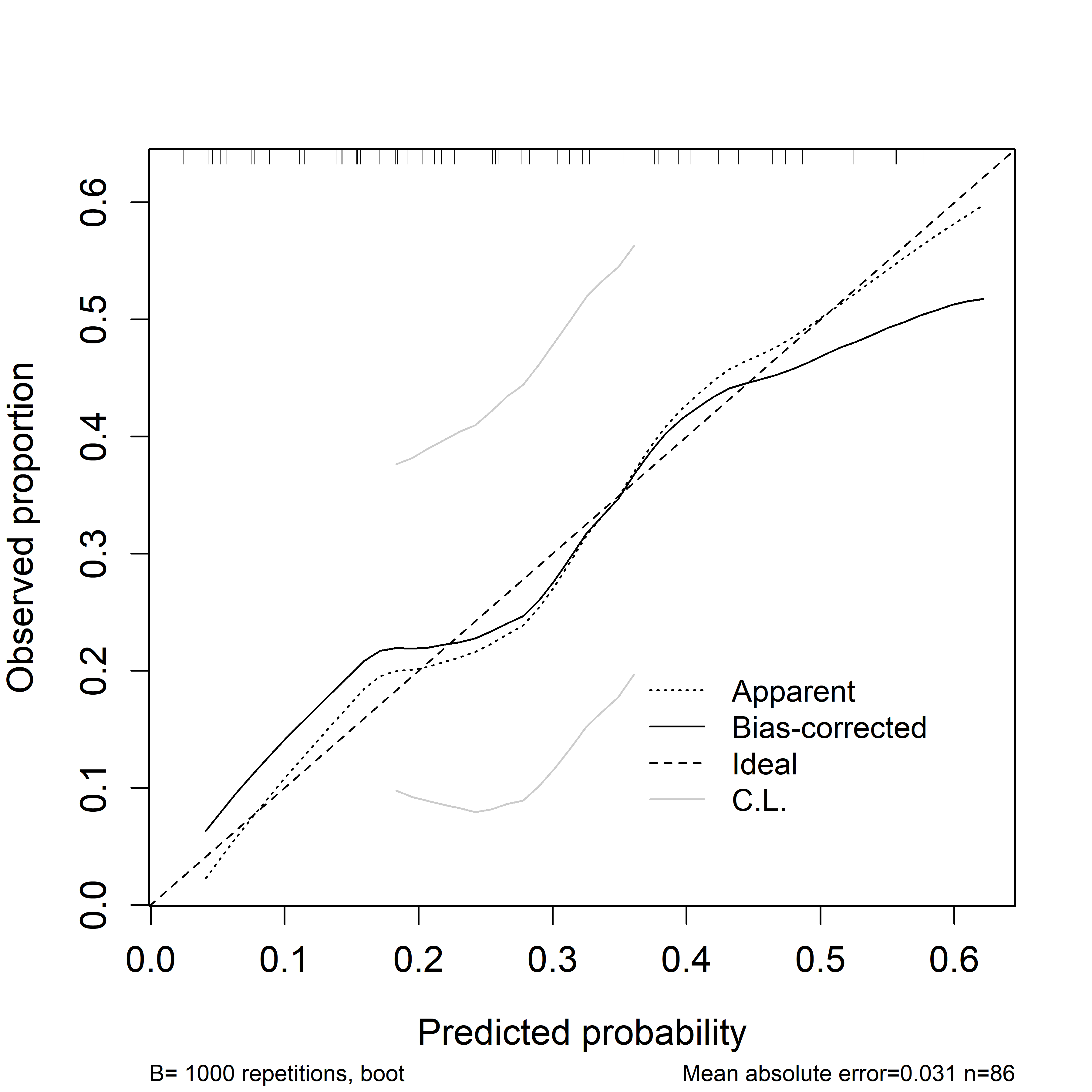

Supplement: Supplementary file 2 [file Image2.tif]
