# Supplementary material for: Higher admission serum total carbon dioxide is independently associated with early seizure recurrence in children with benign convulsions with mild gastroenteritis
Source: Front Pediatr. 2026 Jun 16;14:1884706. doi: 10.3389/fped.2026.1884706 (PMC13314874; doi:10.3389/fped.2026.1884706)
Supplement: Supplementary file 3 [file Supplementaryfile1.docx]

**Supplementary Figure S1.** Bland-Altman plot for the agreement between serum total carbon dioxide (TCO₂) and directly measured bicarbonate (HCO₃⁻) in 29 patients with paired measurements.

The solid line represents the mean difference (0.71 mmol/L), and the dashed lines represent the 95% limits of agreement (−1.37 to 2.79 mmol/L). The dotted line at zero represents the line of perfect agreement. Note the presence of one extreme outlier (difference = −4.70 mmol/L); when this single observation was excluded in sensitivity analysis, the limits of agreement narrowed substantially (0.47 to 1.33 mmol/L), as detailed in the text.

**Supplementa**ry Figure S2. Bootstrap calibration plot for the final multivariable logistic regression model (M3) predicting seizure recurrence within 48 hours.

The plot was generated using 1,000 bootstrap resamples to assess internal validation. The dashed diagonal line represents ideal calibration (where predicted probability perfectly matches observed proportion). The dotted line represents the apparent calibration accuracy, and the solid line represents the optimism-corrected (bias-corrected) calibration curve. The tick marks at the top (rug plot) indicate the distribution of the predicted probabilities in the cohort. Mean absolute error = 0.031.

Supplementary Table S1. Full logistic regression models for seizure recurrence within 48 hours in children with benign convulsions with mild gastroenteritis.

| **Model** | **Predictor** | **OR (95% CI)** | **P value** |
| --- | --- | --- | --- |
| **Model 1 (M1): TCO₂ only (n = 86)** | | | |
| M1 | TCO₂, per 1 mmol/L | 1.169 (1.017-1.345) | 0.028 |
| M1 | Omnibus P | 0.020 |  |
| M1 | Hosmer-Lemeshow P | 0.614 |  |
| M1 | Nagelkerke R2 | 0.089 |  |
| M1 | AUC (95% CI) | 0.692 (0.570-0.813) |  |
| **Model 2 (M2): TCO₂ + seizure variables + sedatives (n = 86)** | | | |
| M2 | TCO₂, per 1 mmol/L | 1.158 (1.005-1.335) | 0.043 |
| M2 | Pre-arrival seizure count, per 1 | 1.240 (0.531-2.895) | 0.619 |
| M2 | Seizure-to-sampling interval, per min | 0.998 (0.991-1.004) | 0.498 |
| M2 | Midazolam (0/1) | 1.447 (0.312-6.698) | 0.637 |
| M2 | Phenobarbital (0/1) | 0.652 (0.204-2.076) | 0.469 |
| M2 | Omnibus P | 0.156 |  |
| M2 | Hosmer-Lemeshow P | 0.447 |  |
| M2 | Nagelkerke R2 | 0.129 |  |
| M2 | AUC (95% CI) | 0.721 (0.606-0.836) |  |
| **Model 3 (M3, final): TCO₂ + age + sedatives (n = 86)** | | | |
| M3 | TCO₂, per 1 mmol/L | 1.235 (1.053-1.448) | 0.009 |
| M3 | Age, per month | 0.900 (0.826-0.980) | 0.016 |
| M3 | Midazolam (0/1) | 1.782 (0.429-7.407) | 0.426 |
| M3 | Phenobarbital (0/1) | 0.571 (0.172-1.900) | 0.361 |
| M3 | Omnibus P | 0.003 |  |
| M3 | Hosmer-Lemeshow P | 0.572 |  |
| M3 | Nagelkerke R2 | 0.243 |  |
| M3 | AUC (95% CI) | 0.762 (0.652-0.871) |  |
| M3 | Bootstrap optimism-corrected AUC | 0.710 |  |
| **Extended model (M_extended): M3 + seizure variables (n = 86)** | | | |
| M_extended | TCO₂, per 1 mmol/L | 1.254 (1.062-1.482) | 0.008 |
| M_extended | Age, per month | 0.891 (0.815-0.974) | 0.011 |
| M_extended | Pre-arrival seizure count, per 1 | 1.341 (0.574-3.131) | 0.498 |
| M_extended | Seizure-to-sampling interval, per min | 0.996 (0.989-1.003) | 0.274 |
| M_extended | Midazolam (0/1) | 1.488 (0.304-7.288) | 0.624 |
| M_extended | Phenobarbital (0/1) | 0.587 (0.170-2.020) | 0.398 |
| M_extended | Omnibus P | 0.009 |  |
| M_extended | Hosmer-Lemeshow P | 0.743 |  |
| M_extended | Nagelkerke R2 | 0.263 |  |
| M_extended | AUC (95% CI) | 0.776 (0.675-0.877) |  |
| **Exploratory model (M_exploratory): M2 + chloride + IV fluids (n = 86)** | | | |
| M_exploratory | TCO₂, per 1 mmol/L | 1.192 (1.021-1.391) | 0.026 |
| M_exploratory | Pre-arrival seizure count, per 1 | 1.956 (0.724-5.285) | 0.186 |
| M_exploratory | Seizure-to-sampling interval, per min | 1.000 (0.994-1.007) | 0.933 |
| M_exploratory | Midazolam (0/1) | 2.369 (0.453-12.390) | 0.307 |
| M_exploratory | Phenobarbital (0/1) | 0.577 (0.176-1.897) | 0.365 |
| M_exploratory | Chloride, per 1 mmol/L | 0.953 (0.809-1.123) | 0.565 |
| M_exploratory | IV fluids before sampling (0/1) | 0.088 (0.009-0.890) | 0.040 |
| M_exploratory | Omnibus P | 0.034 |  |
| M_exploratory | Hosmer-Lemeshow P | 0.854 |  |
| M_exploratory | Nagelkerke R2 | 0.235 |  |
| M_exploratory | AUC (95% CI) | 0.767 (0.660-0.875) |  |
| **Sensitivity analysis: M3 excluding pre-sampling IV fluids (n = 70)** | | | |
| Sens | TCO₂, per 1 mmol/L | 1.328 (1.100-1.603) | 0.003 |
| Sens | Age, per month | 0.892 (0.814-0.977) | 0.014 |
| Sens | Midazolam (0/1) | 3.046 (0.483-19.219) | 0.236 |
| Sens | Phenobarbital (0/1) | 0.643 (0.182-2.278) | 0.494 |
| Sens | Omnibus P | 0.002 |  |
| Sens | Hosmer-Lemeshow P | 0.434 |  |
| Sens | Nagelkerke R2 | 0.306 |  |

Notes: Odds ratios (ORs) are reported per 1-unit increase for continuous predictors (TCO₂ in mmol/L, age in months, seizure-to-sampling interval in minutes, chloride in mmol/L). Midazolam and phenobarbital are indicator variables (0/1), with no anticonvulsant medication as the reference category. IV fluids indicates intravenous fluid administration prior to blood sampling (0/1). AUC values are from ROC analyses in the full cohort unless otherwise specified. Bootstrap optimism-corrected AUC was derived from bootstrap internal validation (1,000 resamples).

Abbreviations: AUC, area under the receiver operating characteristic curve; BE, base excess; CI, confidence interval; IV, intravenous; OR, odds ratio; TCO₂, total carbon dioxide.

Supplementary Table S2. Pearson correlations among serum TCO₂, blood gas bicarbonate, and blood gas parameters in patients with paired blood gas measurements (n = 29).

| **Variable** | **TCO₂** | **HCO₃⁻** | **pH** | **PCO₂** | **BE** | **Lac** |
| --- | --- | --- | --- | --- | --- | --- |
| TCO₂ | 1.000 | 0.966 (<0.001) | 0.193 (0.316) | 0.821 (<0.001) | 0.883 (<0.001) | -0.181 (0.347) |
| HCO₃⁻ | 0.966 (<0.001) | 1.000 | 0.267 (0.161) | 0.807 (<0.001) | 0.944 (<0.001) | -0.177 (0.359) |
| pH | 0.193 (0.316) | 0.267 (0.161) | 1.000 | -0.346 (0.066) | 0.564 (0.001) | 0.093 (0.633) |
| PCO₂ | 0.821 (<0.001) | 0.807 (<0.001) | -0.346 (0.066) | 1.000 | 0.574 (0.001) | -0.235 (0.220) |
| BE | 0.883 (<0.001) | 0.944 (<0.001) | 0.564 (0.001) | 0.574 (0.001) | 1.000 | -0.124 (0.523) |
| Lac | -0.181 (0.347) | -0.177 (0.359) | 0.093 (0.633) | -0.235 (0.220) | -0.124 (0.523) | 1.000 |

Values are Pearson correlation coefficients (two-tailed P values in parentheses). Blood gas subset size was n = 29.

Abbreviations: BE, base excess; Lac, lactate; PCO₂, partial pressure of carbon dioxide; TCO₂, total carbon dioxide.

Supplementary Table S3. Blood gas parameters by seizure recurrence status in the blood gas subset (n = 29).

| **Variable** | **No recurrence (n=15)**  **Mean rank** | **Recurrence (n=14)**  **Mean rank** | **U** | **P value** | **Exact P value** |
| --- | --- | --- | --- | --- | --- |
| pH | 15.57 | 14.39 | 96.5 | 0.710 | 0.715 |
| PCO₂ | 14.00 | 16.07 | 90.0 | 0.511 | 0.533 |
| BE | 14.87 | 15.14 | 103.0 | 0.930 | 0.949 |
| Lac | 15.43 | 14.54 | 98.5 | 0.776 | 0.780 |

Group comparisons were performed using the Mann-Whitney U test. P values are two-tailed.

Abbreviations: BE, base excess; Lac, lactate; PCO₂, partial pressure of carbon dioxide.
